# Monitoring and evaluation of the immune status of female Kunming mice maintained in different biosafety level laboratories

**DOI:** 10.1242/bio.035006

**Published:** 2018-11-07

**Authors:** Lei Guo, Yuan He, Heng Li, Yong Chen, Fanli Zhu, Mengli Yang, Chengyun Yang, Qing Dai, Haijing Shi, Longding Liu

**Affiliations:** Department of respiratory infection, Kunming National High-level Biosafety Research Center, Institute of Medical Biology, Chinese Academy of Medical Science, Kunming, Yunnan 650118, China

**Keywords:** Biosafety, ABSL-2/3/4, Mouse housing, Immune status

## Abstract

High-level biosafety laboratories (BSL), such as BSL-3 and BSL-4, which deal with high infectivity and virulence pathogens, have become indispensable. Mice are frequently used in animal BSL (ABSL) to establish animal models for infection and to evaluate *in vivo* immune responses. A project of monitoring and evaluation on the physiology and immune status of mice housed in different ABSL labs was performed in the ABSL-2/3/4 labs of Kunming National High-level Biosafety Research Center, China. Female Kunming mice were housed in the ABSL-2/3/4 labs for 1 month, and mouse behavior, body physiology/immune status, pulmonary immune status and respiratory bacteria composition were evaluated and compared among mice from the different labs. Mice settled in their new housing environment of the different labs after transfer and gained weight steadily. Blood hematology testing, serum cytokine/chemokine profiles and blood/spleen lymphocyte constitutions were comparable between the ABSL-2/3/4 labs. The numbers of different pulmonary leukocytes in the bronchoalveolar lavage fluid were at baseline levels in mice from the ABSL-2/3/4 labs. Diversity and dominance of mice respiratory bacteria were semblable among the ABSL-2/3/4 labs. Our results confirm the stability of physiology and immune status of Kunming mice maintained in different ABSL-2/3/4 labs for at least 1 month.

## Abstract (precis)

**Summary:** Mice maintained in ABSL-3 and ABSL-4 labs are under normal and stable physiology and immune status.

## INTRODUCTION

Nowadays, biosafety in microbiological and biomedical laboratories has become a criterion for experimental practice worldwide. The biosafety level of a laboratory (BSL) is specified as level 1 to 4, with 4 being the highest safety level, according to the level of severity of human disease caused by pathogens, the transmission of infection from one individual to another, and the degree of protection necessary for personnel and the environment (U.S. Department of Health and Human Services, 2009). BSL-1 and BSL-2 labs are common facilities and can handle pathogens that cause human or animal disease but are unlikely to be a serious hazard to humans and livestock. BSL-3 labs handle pathogens associated with serious or lethal human or animal diseases, usually spread by respiratory contact. BSL-4 labs deal with pathogens that cause serious/lethal human or animal diseases and can be readily transmitted to others and the community; they are in areas where preventive or therapeutic interventions are not usually available. Compared to a BSL-2 lab, a BSL-3 lab is constructed with a ventilation system to keep negative air pressure levels, which draws air into the laboratory from ‘clean’ areas toward ‘potentially contaminated’ areas. The exhaust air should be HEPA (high efficiency particulate air filter) filtered. Biosafety cabinets (BSC) are required to manipulate infectious materials in BSL-3 labs. Personal protective equipment (PPE), such as protective laboratory clothing and respiratory protection (respirator or N95/99 filter mask), is required. A BSL-4 lab is built with stricter protection compared to BSL-3 labs. BSL-4 laboratories are generally set up to be either cabinet laboratories or protective-suit laboratories. In cabinet laboratories, all work must be done within a class III BSC. While in a protective-suit laboratory, all work must be done in a class II BSC by personnel wearing a PPE with full-body, positive-pressure suit supplied with a HEPA-filtered respirator. A BSL-4 lab is pressure-tight with negative pressure, a dedicated supply and exhaust vacuum and decontamination systems as well.

Based on the biosafety level, biosafety laboratories that carry out animal work are classified as animal biosafety level 1-4 (ABSL-1, -2, -3 or -4). Workers in ABSL-3 and ABSL-4 labs need to pay additional attention to safety protection measures for animal isolation, husbandry and zoological experimental procedures. For mouse experimentation, airtight individual ventilated cage (IVC) systems are used to maintain mice, and these systems help reduce contamination from mouse to mouse, mouse to manipulator, and mouse to environment. Besides requiring PPE and mouse-handling procedures (especially those using sharp instruments), ABSL-3/4 labs are strictly specified to protect experimenters. In addition to the effort required to protect the manipulators in ABSL-3/4 labs, the effects of the surrounding environment on the well-being and on the physiological and immunological status of the mice being housed, handled and sampled in the isolated and negative-pressure high-level biosafety lab need to be confirmed. Factors such as mouse housing conditions, sampling procedures, environmental air pressure and air changes, room temperature/humidity/light/noise/vibration, etc., affect mouse behavior and physiology (Balcombe, 2006; Balcombe et al., 2006; Gärtner et al., 1980; Geertsema and Lindsell, 2015; Memarzadeh et al., 2004; Nevalainen, 2014). Thus, this work commits to monitoring and evaluating the physiology and immune status of mice maintained in the ABSL-2/3/4 labs of the Kunming National High-level Biosafety Research Center, China. The effort will contribute to developing etiological and immunological analysis of mouse infection in high-level biosafety labs.

## RESULTS

### Mice maintained in the ABSL-2/3/4 labs

**Fig. 1.**
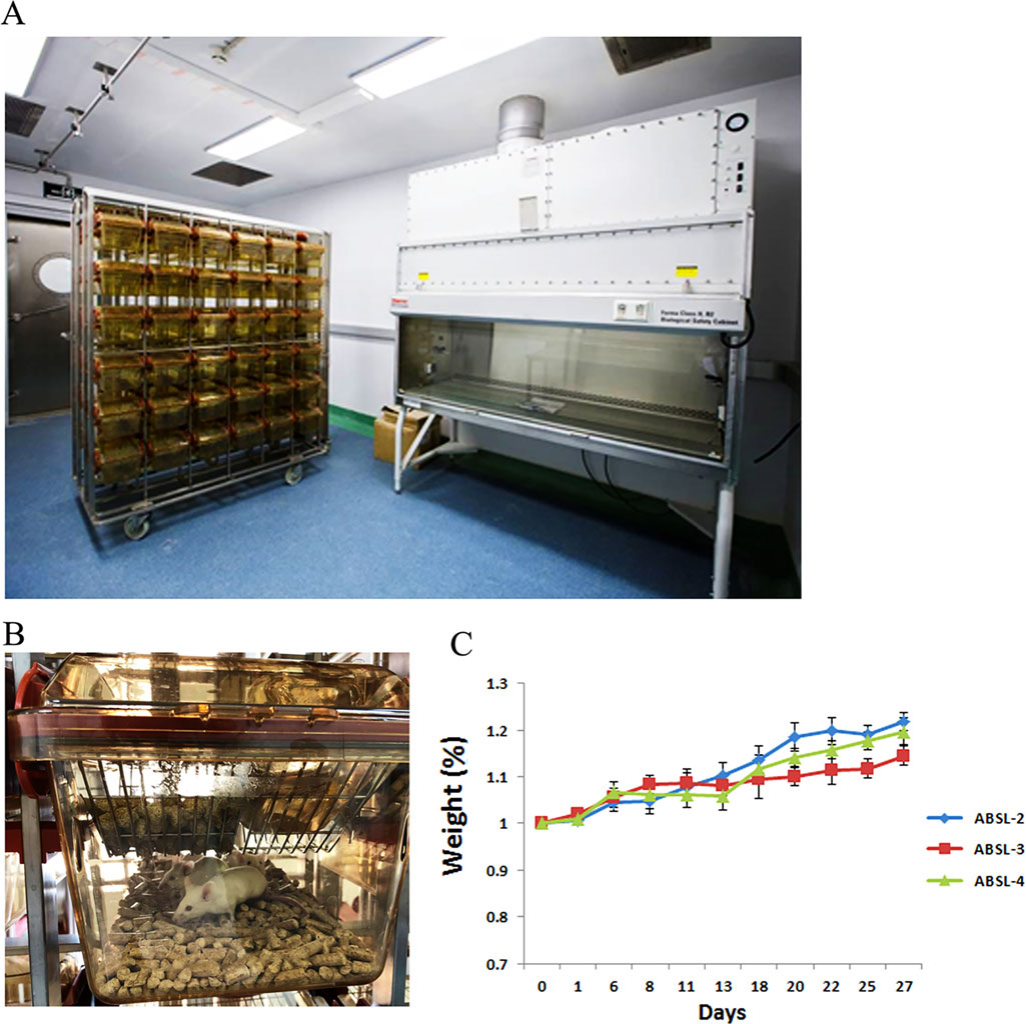
**Mice housed in ABSL-2, ABSL-3, and ABSL-4 labs.** 5- to 6-week-old female KM mice were housed in ABSL-2, ABSL-3, and ABSL-4 labs (*n*=28). (A) An IVC system was used for maintaining mice, and sampling was carried out in a class II BSC, as shown in this picture from an ABSL-3 lab. (B) Mice were housed in an IVC and provided with *ad libitum* food, autoclaved water and compressed wood chip bedding, as shown in this picture. (C) Body weight gain (*n*=16) of the mice was monitored for 1 month. The error bars represent the standard deviation of the total mice in each group.

Mice transferred into the ABSL-2, ABSL-3, and ABSL-4 labs on day 0 settled into their new housing environments the next day, except for the mice from two cages – one located in the ABSL-2 lab and one in the ABSL-4 lab. Fighting with cage mates and wounds on one or two mice were observed in these two cages. To ensure accurate data, these two cage mice were removed from our experiment. Notably, conducting mouse experiments in ABSL-3 and ABSL-4 labs should include additional mice in the experimental design because if mice are lost to the experiment after it has begun, as happened in our experiment, the addition of more mice to the study would be time consuming, as re-evaluation and re-authorization of the amended protocol would need to be done by the biosafety committee and new mice would need to acclimate to the new environment. No aggression or fighting wounds were observed after this point in the mice throughout the month of monitoring. All mice settled themselves at the rear part of the cages, and eating/drinking/defecating occurred at the front of the cages. Grooming was also observed in each cage. Growth curves were drawn for all the ABSL lab mice for the 4 weeks, and those curves show that the mice from each biosafety lab gained weight steadily (Fig. 1C).

### Physiology and immune status of the mice maintained in the ABSL-2/3/4 labs

**Fig. 2.**
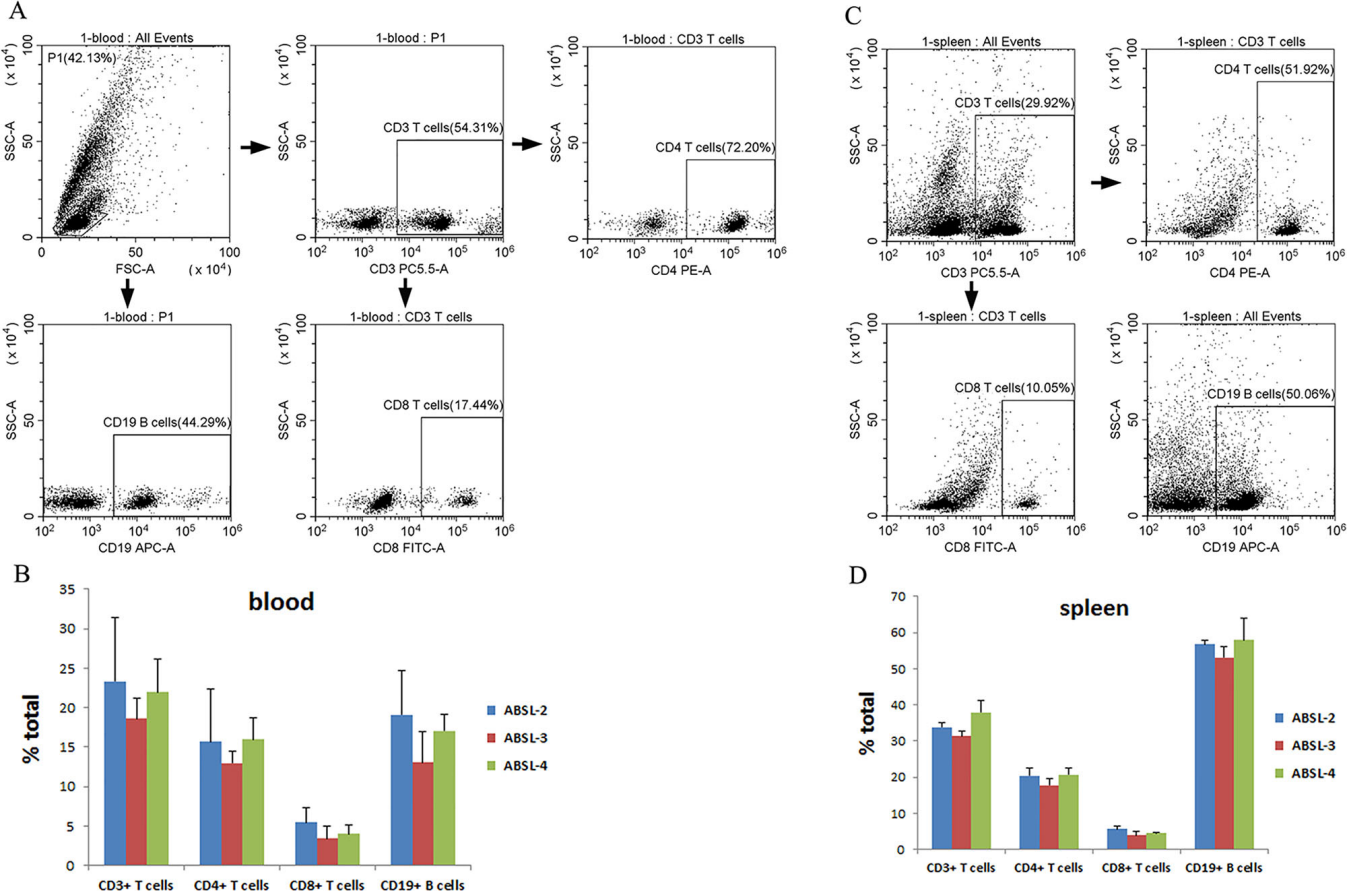
**Constitution of the lymphocyte subtypes of mouse blood and spleen samples.** Mice from the ABSL-2/3/4 labs were euthanized at days 7, 14 and 28 of housing, and blood and spleen samples of each mouse were harvested (*n*=4, each time point and each group). Percentages of CD3+, CD4+, CD8+ and CD19+ lymphocytes from blood (A,B) and spleen (C,D) samples were determined by flow cytometry. (A,C) Sorting illustrations of blood sample and spleen sample. (B,D) Statistics of total percentages of CD3+, CD4+, CD8+ and CD19+ lymphocytes of blood samples and spleen samples in each mouse group. The error bars represent the standard deviation of each mouse group (*n*=12). The *P* value was conducted based on Student's *t*-test.

**Fig. 3.**
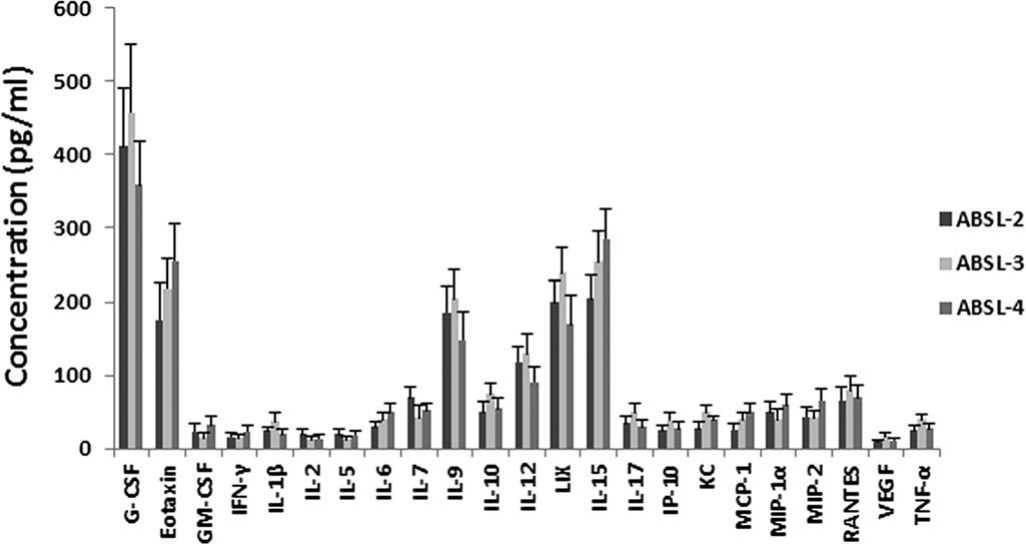
**Multianalyte concentration profile of cytokines and chemokines from mouse serum.** Serum samples were collected as described in the Materials and Methods section from each mouse group housed in the ABSL-2/3/4 labs. Concentrations of multicytokines and chemokines were performed using the Mouse Cytokine/Chemokine Magnetic Bead Panel (Premixed 32 Plex). The error bars represent the standard deviation of each mouse group (*n*=12). The *P* value was determined based on a Student's *t*-test.

Mice were euthanized at the end of weeks 1, 2 and 4 in each biosafety lab group. Blood samples were collected, and hematology tests were performed. Physiological status of mice from each of the ABSL groups during 1 month monitoring were stable and no significant changes were detected (data not shown). No significant difference (*P*<0.05) in the hematology targets was observed between the ABSL-2 and ABSL-3 mice, the ABSL-2 and ABSL-4 mice, and the ABSL-3 and ABSL-4 mice (Table 1). Data collected on the total number of white blood cells (WBC), neutrophils (NE), monocytes (MO), eosinophils (EO) and basophils (BO) suggest that mice in the ABSL-3 and ABSL-4 labs have less standard deviations (s.d.) than those in the ABSL-2 lab (Table 1) with these leukocytes. Lymphocyte subtypes of blood and spleen samples were determined by flow cytometry to further evaluate the immune lymphocyte status of the mice from the different biosafety labs. The results showed that the percentages of blood CD3+, CD4+, CD8+ and CD19+ lymphocytes are similar among the ABSL-2, ABSL-3 and ABSL-4 mouse groups, and no significant differences were found (Fig. 2A,B). Also, the same concordance of CD3+, CD4+, CD8+ and CD19+ percentages in the spleen was demonstrated among the ABSL-2/3/4 labs (Fig. 2C,D), with no statistically significant differences. Cytokines and chemokines are involved in the immune response and inflammation. The cytokine/chemokine profile of the mouse serum was screened by the MILLIPLEX mouse MAP assay. As shown in Fig. 3, absolute serum concentrations were assessed for 23 of the 32 cytokine and chemokine analytes; data for nine other analytes could not be obtained because their signals were below the level of detection. The serum levels of the 23 cytokines and chemokines were not significantly different among the groups of ABSL-2/3/4 mice (Fig. 3). Together, these data suggest that mice maintained in the different biosafety level labs for 1 month did not differ significantly from each other in their physiology and circulating immune status.

**Table 1.**
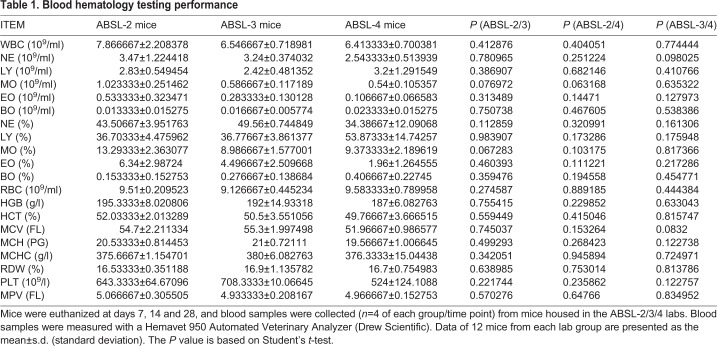
**Blood hematology testing performance**

### Pulmonary immune status and respiratory bacteria composition of the mice maintained in the ABSL-2/3/4 labs

**Fig. 4.**
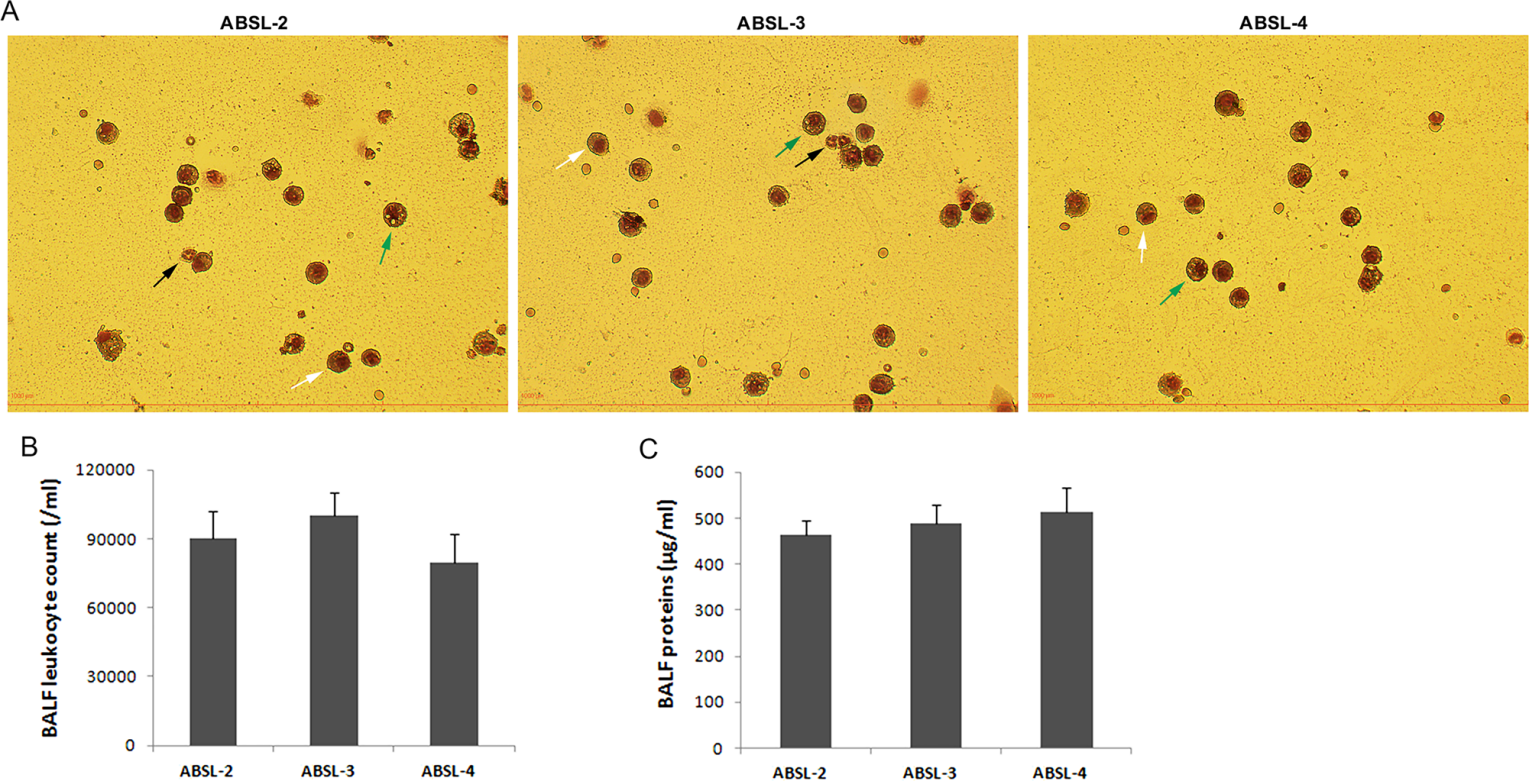
**Lung BALF leukocyte count and protein concentrations.** Lung BALF samples were collected at each mouse euthanasia time point; the total number of BALF leukocytes, BALF leukocyte phenotypes, and total BALF protein concentration was determined for each mouse. (A) Illustrations of BALF leukocyte phenotypes from mice housed in the ABSL-2/3/4 labs under microscope after being cytospun. Black arrow, neutrophils; white arrow, monocytes; green arrow, macrophages (×400 magnifications). (B) Statistics of BALF total leukocyte counts among the ABSL-2, ABSL-3, and ABSL-4 mouse groups. (C) Statistics of BALF total protein concentrations among the ABSL-2, ABSL-3, and ABSL-4 mouse groups. The error bars represent the standard deviation of each mouse group (*n*=12). The *P* value was determined based on a Student's *t*-test.

Moreover, lung BALFs of mice were collected and cell counts, cell phenotype, and protein content was determined to examine whether air pressure levels in the different biosafety rooms impact the pulmonary immune microenvironment. The number of total leukocytes was low in BALF and did not differ significantly among the ABSL-2/3/4 groups (Fig. 4B). Specifically, most immunocyte subsets in BALF are alveolar monocytes and macrophages (Fig. 4A), which serve a surveillance role in the normal mouse lung. Neutrophils are rare in normal BALF, while BALF neutrophilia is a critical marker of a pulmonary inflammatory/immune response (Fig. 4A). Eosinophilia can be indicative of allergic reactions, and few can be seen in the BALF of these mouse groups (Fig. 4A). In addition, analysis of the total protein concentrations in the BALF from the different mouse groups showed that the total BALF protein levels were all at baseline, and no statistically significant differences were observed (Fig. 4C). Thus, the above data suggests that pulmonary immune statuses of the ABSL-2/3/4 mice were all at a normal level.

**Fig. 5.**
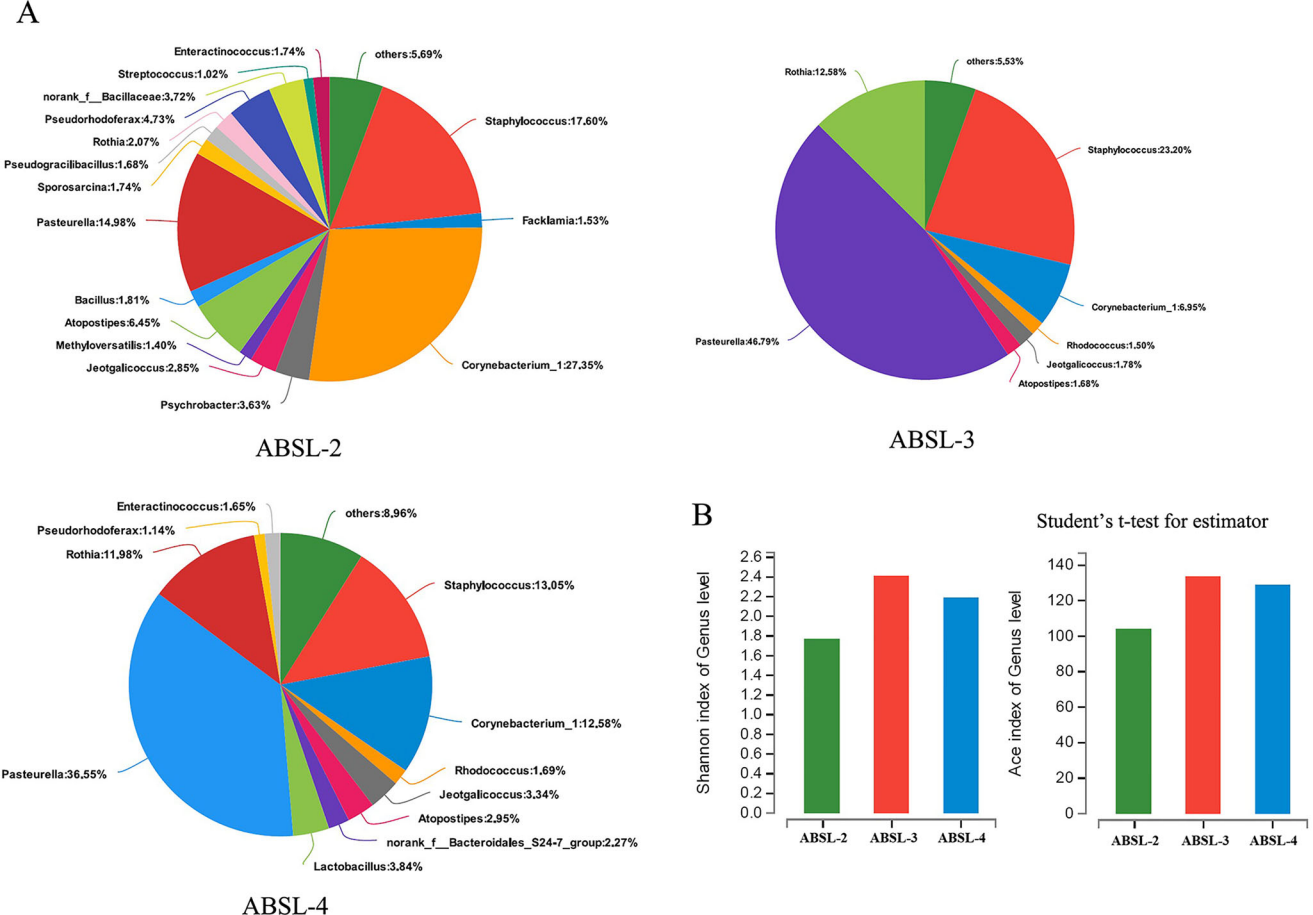
**URT bacteria composition.** Nasopharyngeal lavage samples were collected at each mouse euthanasia time point; total DNA was extracted from each sample for bacterial 16S rRNA amplicons sequencing. (A) Community analysis pieplot on Genus level of the URT bacteria in the each ABSL-2/3/4 mouse groups. (B) Shannon index and abundance-based coverage estimator (ACE) index of Genus level of the URT bacteria microbiota of the ABSL-2/3/4 mouse groups. Student's *t*-test for estimator (*n*=12, each group).

Besides, respiratory microbiota reflects respiratory immunity and respiratory health (Man, et al., 2017; Schenck, et al., 2016; Yan, et al., 2013). The composition of the respiratory bacterial from upper respiratory tract (URT) was investigated by extracting DNA directly from nasopharyngeal lavage for 16S rRNA gene amplification. We used hypervariable regions V1 and V2 to perform phylogenetic discrimination with the barcode primers 27F/338R (Pettigrew, et al., 2012). In total, 35,660±8376 amplicons reads per sample were obtained. On the phylum level, the URT bacteria communities from the ABSL-2/3/4 groups were all composed predominantly of abundant Pasteurella (14.98–46.79%), Staphylococcus (13.05–23.20%) and Corynebacterium (6.95–27.35%) (Fig. 5A). The number of Rothia increased in the ABSL-3/4 groups (11.98–12.58%) compared to the ABSL-2 group (2.07%) (Fig. 5A). The Shannon index showed that the diversity of bacterial microbiota does not differ significantly from each of the ABSL-2/3/4 groups (Fig. 5B). The richness of the bacterial microbiota was also comparable among the ABSL-2/3/4 groups as measured by the abundance-based coverage estimator (ACE), which shows the number of sequences mapping to bacterial genomes (Fig. 5B). Together, these data suggest that the richness and diversity of the respiratory bacterial microbiota composition were relatively stable between the ABSL-2/3/4 groups, which indicates the constant respiratory immune microenvironment of the mice maintained in the ABSL-2/3/4 labs.

## DISCUSSION

Mice maintained in ABSL-3/4 labs were used to establish an infection animal model for severe/lethal or unknown pathogens. Experiments for evaluating the etiology, immunology, pharmacology and vaccinology are often carried out on this model. Usually, the period of an acute infection experiment is no more than one month in ABSL-3/4 labs. The ability of the newly enrolled mice to settle into the isolated and negative pressure ABSL3/4 labs without having their well-being, physiological status and immunological status disturbed for the entire experiment period is essential to the experimental outcomes. Air pressure/speed/temperature/humidity, housing condition/handling and sampling procedures affect mouse well-being and physiology (Balcombe, 2006; Balcombe et al., 2006; Gärtner et al., 1980; Geertsema and Lindsell, 2015; Memarzadeh et al., 2004; Nevalainen, 2014). The air speed/changes or ventilation rate of the IVC could affect mouse physiology when this rate is more than 80 times per hour (Krohn, et al., 2003). Routine cage changing, bedding type and volume, and housing density could affect mouse physiology (Kostomitsopoulos, et al., 2012; Morgan et al., 2014; Reeb-Whitaker, et al., 2001; Rosenbaum, et al., 2009; Sanderson, et al., 2010). Cage changing can transiently alter the intestinal microbiota, which may have profound effects on the host immune response (Ma, et al., 2012; Thoene-Reineke, et al., 2014). Mouse handling also affects the lymphocyte subpopulations and antibody production (Moynihan, et al., 1990, 1992). In the experiments described herein, the room housing temperature, housing humidity, IVC housing environment, mouse maintenance routines and sampling procedures were kept the same among the ABSL-2/3/4 labs. Noises in the ABSL-3/4 lab facilities are controlled at below 55 dB and would not affect the mice, as the mouse hearing range is more extensive than that of humans (Reynolds et al., 2010). The key variable in our experiment is the environmental negative air pressure levels. Mice housed in the different biosafety labs are substantially under the macro-environment negative pressures (room level) and the micro-environment negative pressures (cages of the IVC system). Based on biosafety demands, the room negative air pressure increases with the elevated biosafety laboratory level for securing the release of infectious and contaminated air. The room negative pressures of the ABSL-2, ABSL-3 and ABSL-4 units in our facility were −30±10 pa, −140±10 pa and −170±10 pa, respectively, compared to the local barometric pressures. Although the same IVC system with same ventilation rate setting was used in our ABSL-2/3/4 labs, the air change rates in the IVC cages during their running differed from ABSL-2 and ABSL-3/4 labs (about 60 ACH in the ABSL-2 IVC cages, 50 ACH in the ABSL-3/4 IVC cages). This discrepancy suggests that environmental negative pressure probably influenced the mouse housing environment in our ABSL-2/3/4 labs which may also have impacted mice living there. Based on our data, mice transferred into new IVCs in the ABSL-2, ABSL-3 and ABSL-4 labs all settled in their new housing environments after transfer, with a steady increase in weight occurring thereafter, and no statistically significant difference was observed in the blood physiological index and immunological parameters among the ABSL-2/3/4 groups. In contrast to the ABSL-2 mice, the s.d.s of the total numbers of the blood WBC, NE, MO, EO and BO of the ABSL-3 and ABSL-4 mice were smaller (Table 1), which suggests that mice housed in higher biosafety level labs have more stable leukocyte circulation in the body. This result is possibly due to the undisturbed, isolated experimental environment provided in the ABSL-3/4 labs. In fact, only one experiment is authorized to be conducted in a single ABSL-3 or ABSL-4 lab at a particular time by the biosafety committee under biosafety consideration, and no other experiment would be allowed to proceed at the same time in the same lab. Also, no unauthorized person or irrelevant animal would be allowed to enter or transfer into the lab while the experiment was ongoing. These restrictions contribute to a simple, stable and controllable experimental environment. In an ABSL-2 lab, different mice that are being used for different purposes are commonly housed in one room, and even in one IVC system, with handling and sampling being conducted every day in the same room, which could influence mouse habits and physiology. A wide variety of experimental influences have been suggested that can contribute to problems with reproducibility of research outcomes using rodents (Lewejohann et al., 2006; Toth, 2015). Possibly, the difference between using a class II A2 BSC in the ABSL-2 lab and a class II B2 BSC in the ABSL-3/4 labs for sampling may also contribute to inequality in the samples, as the recirculation airflows inside the class II A2 and B2 BSCs are different; this potential possibility needs to be further investigated. Moreover, evaluations on respiratory infection and immune response are critical experiments in ABSL-3/4 labs and our work demonstrates – for the first time – that housing mice in different biosafety level laboratories does not impact their respiratory immune status for at least 1 month of maintenance.

Overall, our work monitored and evaluated mouse physiological and immunological status in different biosafety level laboratories – ABSL-2/3/4 – for 1 month. According to the data obtained, the physiological and immunological levels of mice maintained in the ABSL-2/3/4 labs were at normal levels for the entire housing period. We recommend that mouse experiments in ABSL-3/4 labs begin after allowing the mice to settle down and acclimate to their new housing environment, for example, 5–7 days would be beneficial to the outcomes of reliable analysis.

## MATERIALS AND METHODS

### Animals

Female Kunming mice (KM mice), aged 5–6 weeks, were used in all experiments, male mice were not used because they are often aggressive and cause wounds to their cage mates. The mice were raised and maintained under specific-pathogen-free (SPF) conditions at the Central Animal Care Services of the Institute of Medical Biology, Chinese Academy of Medical Sciences. The mice were free from infections of mouse hepatitis virus, minute virus of mice, pneumonia virus of mice, *Citrobacter rodentium*, mouse adenovirus, mouse cytomegalovirus, Theiler’s murine encephalomyelitis virus, mouse parvovirus, mouse rotavirus, mouse thymic virus, lymphocytic choriomeningitis virus, Sendai virus, Mycoplasma pulmonis, Salmonella spp, and Helicobacter spp. All animal experiments were conducted with prior approval from the animal ethics committee of the Institute of Medical Biology, Chinese Academy of Medical Science, with permit number [2017] 64, according to the national guidelines on animal studies in China.

### Housing and sampling

Mice were randomly divided and housed in ABSL-2, ABSL-3, and ABSL-4 units (protective-suit laboratory) of the newly built Kunming National High-level Biosafety Research Center. The ABSL-2, ABSL-3 and ABSL-4 units were isolated from each other in the Center, and the model of ABSL-4 is a protective-suit laboratory. The experiment described here was performed under prior approval from the biosafety committee of the Center (permit number, PO-M-4-01/18). The authors Lei Guo and Yuan He, who conducted the mouse husbandry, sampling, and testing in the ABSL-2/3/4 units, were trained first and authorized to enter and perform the experiment in the ABSL-2/3/4 units after passing the exams for ABSL-3 and ABSL-4 access in sequence (Le Duc et al., 2008).

Mice were randomly divided into three equal groups, each of which (*n*=28) was housed in an airtight and negative pressure IVC system (type 21SO60, TECNIPLAST, Italy) in the ABSL-2, ABSL-3, and ABSL-4 units (Fig. 1A,B). The mice were monitored by surveillance camera located in the upper front of the IVC system 24 h a day. All mice were housed in groups of four per cage. The cages were ventilated with HEPA filters at 60 air changes per hour (ACH) in the ABSL-2 lab and 50 ACH in the ABSL-3/4 labs. Mice were kept under steady physical conditions (20–22°C temperature; 40–60% humidity; −30±10 pa negative air pressure) in the ABSL-2 lab on a 12 h:12 h light:dark cycle in the rooms. Based on biosafety demands, the room negative air pressure increases with the elevated biosafety laboratory level for securing the release of infectious and contaminated air. Mice kept in the ABSL-3 lab were maintained under constant conditions (21±1°C temperature; 50±5% humidity; −140±10 pa negative air pressure; <55 dB background noise) on a 12 h:12 h light:dark cycle. The environment in the ABSL-4 was almost the same as in the ABSL-3, except that the negative air pressure was −170±10 pa. Irradiated diet (XIETONG ORGANISM, Co60, China) and autoclaved water were provided for mice *ad libitum*. Approximately 200 g of irradiated compressed wood chip bedding (XIETONG ORGANISM, Co60, China) was provided in each IVC cage. Bedding was changed once a week.

Mice were housed for 1 month in the ABSL-2/3/4 labs, and their behavior and weight were monitored sequentially three times a week. Mice from each ABSL lab were euthanized at the end of weeks 1, 2 and 4 (*n*=4, one cage of each time point/group), and the blood, lung bronchoalveolar lavage fluid (BALF), nasopharyngeal lavage fluid and spleen were sampled in a BSC (Class II, A2, SUJIE PURIFICATION, China) in the ABSL-2 lab and in a BSC (Series Class II, B2, Thermo Fisher Scientific) in the ABSL-3 and ABSL-4 labs. All routine laboratory mouse handling and sampling procedures were exactly the same in every biosafety lab under a standard operation procedure (SOP) formulated by the animal ethics committee of the Institute of Medical Biology, Chinese Academy of Medical Science. *In vitro* assays were conducted using the same equipment maintained in each ABSL facility, equipment such as flow cytometry and centrifuge which can generate aerosols were operated in the BSCs in the ABSL-3 and ABSL-4 labs.

### Cytokine assays

Serum from the blood of each mouse was collected at the time of necropsy and stored at −80°C until analysis. The MILLIPLEX MAP Multiplex Assay System (Merck Millipore, Germany) was used to evaluate the multicytokine profile of the mouse serum. The Mouse Cytokine/Chemokine Magnetic Bead Panel (Premixed 32 Plex) (Cat. No. MCYTMAG-70K-PX32) was introduced to measure the following cytokines: G-CSF (Granulocyte-colony stimulating factor), Eotaxin, GM-CSF (Granulocyte-macrophage colony-stimulating factor), IFN-γ, IL-1β, IL-2, IL-5, IL-6, IL-7, IL-9, IL-10, IL-12p70, LIX (Lipopolysaccharide-induced CXC Chemokine), IL-15, IL-17, IL-10, KC (Keratinocyte-derived cytokine), MCP-1 (Monocyte chemotactic protein 1), MIP-1α (Macrophage inflammatory protein 1α), MIP-2, RANTES (Regulated upon activation, normal t-cell expressed, and secreted), VEGF (Vascular endothelial growth factor) and TNF-α. The assay was performed according to the manufacturer's protocol as previously published (Jeon et al., 2012).

### Flow cytometry

The procedure for the flow cytometry procedure was as described before (Guo et al., 2017). Briefly, blood samples were washed in fluorescence-activated cell sorter buffer after lysis with BD FACS Lysing Solution (BD Biosciences). Cell surface staining was done using Mouse BD Fc Block and anti-mouse CD3-PerCP (Cat. No. 551163, Lot 7038886, clone 145-2C11), CD4-PE (Cat. No. 553652, Lot 7038886, clone H129.19), CD8-FITC (Cat. No. 553030, Lot 7170636, clone 53-6.7), and CD19-APC (Cat. No. 550992, Lot 7081501, clone 1D3) (BD Biosciences). Flow cytometry data were collected on the CytoFLEX (Beckman Coulter, USA) and analyzed using CytoExpert 2.0.

### BALF cells and total proteins

BALF was performed immediately following euthanasia of the mice by cervical dislocation. After the trachea was exposed, the lungs were lavaged four times with 0.8 ml of cold sterile PBS, and the BALF was centrifuged at 1500 ***g*** for 10 min at 4°C. Supernatants were collected, and the total BALF protein was determined by standard Bradford assay (TIANGEN, China) according to the manufacturer's instruction. All leukocytes in the BALF were counted, 200 μl of which was cytospun onto slides, and the cells were stained with the Wright-Giemsa Stain Kit (Nanjing Jiancheng Bioengineering, China) for differential leukocyte identification under a microscope.

### Bacterial 16S rRNA amplicons sequencing

DNA was extracted from nasopharyngeal lavage specimens of the mice, and PCR was performed using hypervariable regions V1 and V2 to perform phylogenetic discrimination with the barcode primers 27F/338R (Pettigrew, et al., 2012). Libraries were pooled and sequenced using an Illumina MiSeq sequencer by the support of Majorbio (Majiorbiogroup BioTech, China). Sequences were assigned to closed-reference operational taxonomic units (OTUs) at a 97% identify threshold using bacterial 16S sequences from the database Silva 128/16S-bacteria database. The OTU data were taken out flat by the smallest effective samples. α diversity was analyzed by mothur and statistical significance was evaluated by Student's *t*-test.

### Statistical analysis

Data are presented as the means±s.d. (standard deviation) of the experiments. Significant differences among the groups were determined using Student's *t*-test (two-tailed, unequal variances) at a significance level of *P*<0.05.
